# Cj1388 Is a RidA Homolog and Is Required for Flagella Biosynthesis and/or Function in *Campylobacter jejuni*

**DOI:** 10.3389/fmicb.2019.02058

**Published:** 2019-09-06

**Authors:** Jessica Irons, Jessica C. Sacher, Christine M. Szymanski, Diana M. Downs

**Affiliations:** ^1^Department of Microbiology, University of Georgia, Athens, GA, United States; ^2^Department of Biological Sciences, University of Alberta, Edmonton, AB, Canada; ^3^Complex Carbohydrate Research Center, University of Georgia, Athens, GA, United States

**Keywords:** RidA, Cj1388, motility, autoagglutination, flagella, 2-aminoacrylate, *Campylobacter jejuni*

## Abstract

*Campylobacter jejuni* is the leading bacterial cause of acute gastroenteritis worldwide and thus significant to public health. *C. jejuni* primarily lives in the gastrointestinal tracts of poultry and can contaminate meat during processing. Despite a small genome, the metabolic plasticity of *C. jejuni* allows proliferation in chicken ceca and mammalian host intestines, and survival in environments with a variety of temperatures, pH, osmotic conditions, and nutrient availabilities. The exact mechanism of *C. jejuni* infection is unknown, however, virulence requires motility. Our data suggest the *C. jejuni* RidA homolog, Cj1388, plays a role in flagellar biosynthesis, regulation, structure, and/or function and, as such is expected to influence virulence of the organism. Mutants lacking *cj1388* have defects in motility, autoagglutination, and phage infectivity under the conditions tested. Comparison to the RidA paradigm from *Salmonella enterica* indicates the phenotypes of the *C. jejuni cj1388* mutant are likely due to the inhibition of one or more pyridoxal 5′-phosphate-dependent enzymes by the reactive enamine 2-aminoacrylate.

## Introduction

The Rid/YER057c/UK114 protein superfamily (COG0251) is broadly conserved throughout all domains of life (Kim et al., 2001; Leitner-Dagan et al., 2006; Lambrecht et al., 2012, 2013; Downs and Ernst, 2015; Niehaus et al., 2015; ElRamlawy et al., 2016). Based on phylogenetic analysis, the superfamily was divided into eight subfamilies; RidA, which includes homologs of the archetypical protein from *Salmonella enterica*, and Rid1-7, which are not well understood (Niehaus et al., 2015). Prokaryotic genomes can encode several members of the Rid1-7 subfamilies while also encoding one or more RidA proteins. In many genomes, the RidA homologs are not annotated with the ascribed biochemical function for these proteins. The RidA, r eactive i ntermediate d eaminase A, of *S. enterica* was found to be an enamine deaminase, and multiple homologs from the three domains of life have similar activity (Lambrecht et al., 2010; Lambrecht et al., 2012; Niehaus et al., 2015; ElRamlawy et al., 2016; Ernst and Downs, 2018). In some organisms, a cellular role for RidA involves quenching the reactive metabolite 2-aminoacrylate (2AA) to prevent damage to specific pyridoxal 5′-phosphate (PLP)-dependent enzymes (Schmitz and Downs, 2004; Christopherson et al., 2008; Flynn and Downs, 2013; Flynn et al., 2013; Lambrecht et al., 2013; Ernst et al., 2014, 2016; Niehaus et al., 2015; Irons et al., 2018). RidA homologs have a similar role in at least *Escherichia coli*, *Pseudomonas aeruginosa*, and *Saccharomyces cerevisiae*, although the phenotypic consequences of a *ridA* mutation depends on the specific metabolic network architecture of the organism (Borchert and Downs, 2017b; Ernst and Downs, 2018; Irons et al., 2018). Enzyme damage resulting from accumulated 2AA can impact growth, motility, biofilm formation, iron homeostasis, and potentially virulence.

*Campylobacter jejuni* NCTC 11168, a prominent diarrheal pathogen, encodes two members of the Rid superfamily, a RidA homolog (Cj1388*)*, and a protein from the Rid2 subfamily (Cj0327). Data presented herein confirmed Cj1388 is a RidA protein and for clarity this locus is designated *_cj_ridA* throughout. Previous data suggested that *C. jejuni* Cj1388 (_Cj_RidA) plays a role in flagella-flagella interactions, possibly through regulation of flagellar glycan modification (Reuter et al., 2015). Additionally, _Cj_RidA has been highlighted in several global -omics studies in *C. jejuni* strains 11168 and 81176 (strain specific gene designation *cj1388* or *cj1390*, respectively) (Woodall et al., 2005; Reid et al., 2008; Taveirne et al., 2013; Clark et al., 2014; Flint et al., 2014; Guccione et al., 2017; Hao et al., 2017). These data sets suggest _Cj_RidA could play a direct or indirect role in virulence, antibiotic resistance, acid adaptation, growth with bile salts, and hydrogen peroxide and oxygen stress. Finally, *_cj_ridA* is a member of the HeuR (He me u tilization r egulator) regulon (Reuter et al., 2015; Johnson et al., 2016). HeuR is a PAS-domain containing regulator, and thought to be regulated in response to changing environmental cues (Reuter et al., 2015; Johnson et al., 2016). In other *Campylobacter* spp. including *C. coli*, *C. upsaliensis*, and *C. lari*, there is a co-occurrence of *heuR* and *_cj_ridA* in the genome, suggesting *_cj_ridA* may be regulated in response to differing environmental conditions in these organisms.

The _Cj_RidA has been consistently misannotated as an endoribonuclease in *C. jejuni* studies, obscuring its likely connection to PLP-dependent enzymes and metabolism. In *S. enterica*, RidA catalyzes the hydrolysis of the 2AA intermediate formed by several PLP-dependent enzymes (Lambrecht et al., 2012; Ernst et al., 2014, 2016). In the absence of RidA, free 2AA accumulates and can covalently inactivate certain PLP-dependent enzymes such as serine hydroxymethyltransferase (SHMT) (GlyA; EC 2.1.2.1), alanine racemases (Alr/DadX; EC 5.1.1.1), and transaminase B (IlvE; EC 2.6.1.42), leading to defects in one-carbon unit metabolism, cell-wall synthesis, and isoleucine biosynthesis, respectively (Schmitz and Downs, 2004; Flynn et al., 2013; Ernst and Downs, 2016). The activity of the enzymes targeted by 2AA can be decreased by 30–50% in strains lacking RidA (Lambrecht et al., 2010; Ernst and Downs, 2016, 2018; Borchert and Downs, 2017b; Irons et al., 2018). To date, the diverse phenotypes of organisms lacking RidA suggest that there are additional and unknown targets of 2AA, possibly extending beyond PLP-dependent enzymes.

In at least *S. enterica*, *E. coli*, *P. aeruginosa*, *S. cerevisiae*, and *Arabidopsis thaliana*, a biosynthetic threonine/serine dehydratase (IlvA), acting on serine is the main source of 2AA in *ridA* mutants (Lambrecht et al., 2012; Niehaus et al., 2014; Borchert and Downs, 2017a; Ernst and Downs, 2018; Irons et al., 2018). In each of these organisms, IlvA has a regulatory domain and is allosterically inhibited by isoleucine (Gallagher et al., 1998; Schmitz and Downs, 2004). As a consequence, the presence of isoleucine eliminated generation of 2AA and suppressed the phenotypes of a *ridA* mutant. The specific targets of 2AA that result in detectable defects vary in different organisms. In *S. enterica*, 2AA accumulation causes a growth defect reversed by exogenous glycine; in *E. coli* 2AA accumulation-induced growth inhibition was reversed by exogenous aspartate, or purines; in *P. aeruginosa*, 2AA accumulation is detrimental to growth and partially reversed by exogenous proline and polyamines; and in *S. cerevisiae* mitochondrial accumulation of 2AA leads to loss of mitochondrial DNA and reduced heme biosynthesis (Flynn et al., 2013; Ernst and Downs, 2016, 2018; Borchert and Downs, 2017b; Irons et al., 2018) (Whitaker and Downs, unpublished). The diverse effects of 2AA emphasize the complexity of the metabolic network and our limited understanding of the integration between biochemical pathways.

This study was initiated to understand the physiological role of _Cj_RidA (Cj1388) in *C. jejuni* 11168. Although the levels of *_cj_ridA* were noted in multiple global studies, this gene was peripheral in those studies, and in many cases the results were not verified nor was the effect determined to be direct or indirect. The data herein suggest a role for _Cj_RidA in flagellar biosynthesis, structure, glycosylation, and/or function. Further, we confirmed that _Cj_RidA and the Rid2 subfamily member Cj0327, have enamine deaminase activity *in vivo* and *in vitro*. This work extends the list of organisms known to encode functional enamine deaminases of the Rid family.

## Results

### *Cj1388* and *Cj0327* Deaminate 2-Aminoacrylate *in viv*o

**FIGURE 1:**
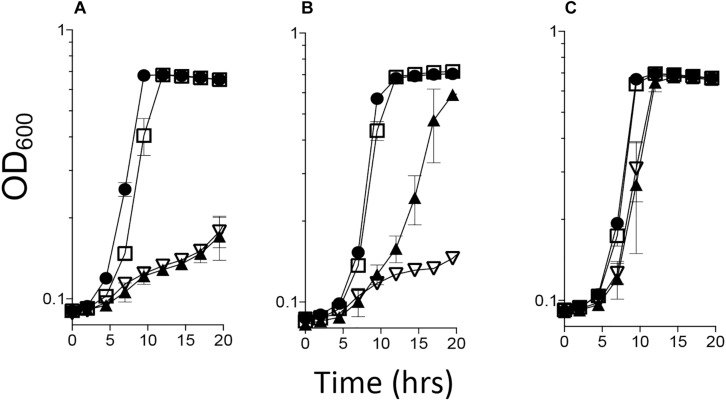
*_cj_ridA* complements a *Salmonella enterica ridA* mutant. A *S. enterica ridA* mutant with one of four plasmids was grown in a 96-well plate at 37°C shaking in minimal glucose (11 mM) medium with: **(A)** serine (5 mM), **(B)** serine and arabinose (0.2%), or **(C)** serine, arabinose and isoleucine (1 mM). The *S. enterica* strain carried plasmids expressing *S. enterica ridA* (filled circles), *C. jejuni ridA* (*_cj_ridA*, open squares), *C. jejuni rid*2 (*cj0327*, closed triangles), or empty vector (open triangles).

A *S. enterica ridA* mutant fails to grow on minimal medium with serine due to the accumulation of 2AA that is generated by the biosynthetic serine/threonine dehydratase encoded by *ilvA* (EC 4.3.1.19) (Schmitz and Downs, 2004; Lambrecht et al., 2013). A *S. enterica ridA* mutant was transformed with pBAD24 constructs harboring a gene encoding Rid proteins from *C. jejuni* (*_cj_1388*/*_cj_ridA* or *cj0327*), the *S. enterica ridA* (*_se_ridA*) under the control of an arabinose promoter, or an empty vector control. Growth was monitored in minimal glucose medium with 5 mM serine and the data are shown in Figure 1. Plasmids carrying either *_cj_ridA* (pDM1577) or *_se_ridA* (pDM1439) restored full growth to the *S. enterica ridA* mutant without inducing expression of the plasmid encoded genes. In contrast, *cj0327* (pDM1588) partially restored growth, and only when its expression was induced with arabinose.

The inability of Cj0327, a Rid2 subfamily member, to fully complement the *S. enterica ridA* mutant was consistent with the results obtained for proteins annotated as Rid1-3 from *P. aeruginosa*, *Yersinia pestis*, *E. coli*, *Acinetobacter baylyi*, and *Pseudomonas syringae* (Hodge-Hanson and Downs, 2017; Irons et al., 2018) (Irons et al., unpublished). The partial complementation by Cj0327 and other proteins from the Rid1, 2, and 3 subfamilies suggests that these proteins may deaminate primarily non-2AA enamines *in vivo*. The specific physiological role of Cj0327 was not pursued further here.

### *Cj1388* and *Cj0327* Have Deaminase Activity *in vitr*o

**FIGURE 2:**
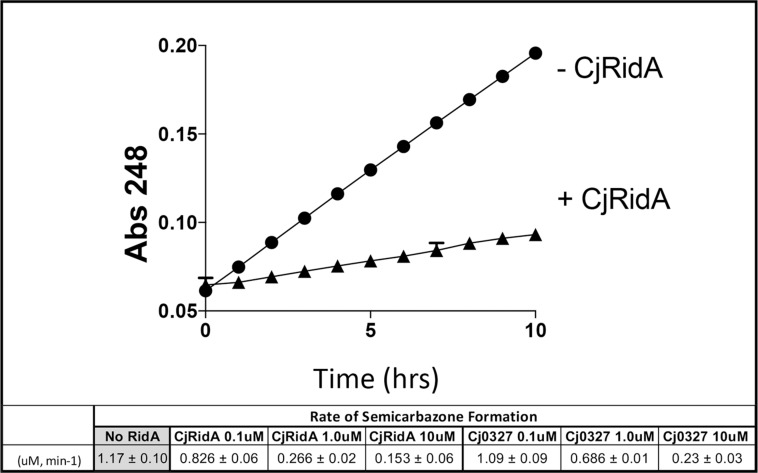
_Cj_RidA and Cj0327 are imine deaminases *in vitro*. Each reaction mixture contained potassium pyrophosphate (50 mM, pH 8.7), neutralized semicarbazide (10 mM), bovine liver catalase (1 μg), and L-amino acid oxidase (0.5 μg) with or without the addition of _Cj_RidA or Cj0327. 2-aminobutyrate (0.5 mM) was added to start the reaction and absorbance at 248 nm was monitored for 10 min. The graph shows the absorbance over time for reactions without _Cj_RidA (circles) or with _Cj_RidA at a final concentration of 1.0 μM (triangles). Error bars represent standard deviation of the mean determined from three technical triplicates by GraphPad Prism 7.0c. The molar extinction coefficient for semicarbazone (ε = 10,300 M^–1^ cm^–1^) was used to calculate the rate of semicarbazone formation (μM, min-1) in reactions without Rid proteins and with _Cj_RidA and Cj0327 in concentrations of 0.1, 1.0, and 10 μM. Standard deviation of the mean was determined from three technical triplicates by GraphPad Prism 7.0c.

L-amino acid oxidase (LOX or LAAO)-based assays were used to assess the ability of purified Cj1388 and Cj0327 to deaminate imines *in vitro* (Hafner and Wellner, 1979; Niehaus et al., 2014; Niehaus et al., 2015; Hodge-Hanson and Downs, 2017; Degani et al., 2018). 2-aminobutyrate was provided as substrate, resulting in the LOX-dependent formation of 2-iminobutyrate. This imine reacts with semicarbazide to produce a semicarbazone which is monitored at 248 nm. Rid proteins can compete for the imine, converting it to the ketoacid, 2-ketobutyrate, similar to a reaction RidA catalyzes *in vivo*. Thus in this assay, the rate of semicarbazone formation is inversely proportional to Rid activity. The rate of semicarbazone formation (μM, min^–1^) with the addition of Cj1388 (_Cj_RidA) or Cj0327 is shown in Figure 2. Consistent with the *in vivo* complementation data in an *S. enterica ridA* mutant, _Cj_RidA has greater deaminase activity than Cj0327. When the Rid proteins are provided at higher concentrations (i.e., 10 μM), semicarbazone formation is reduced to the same extent by each protein, consistent with a saturating concentration of enzyme.

### *Campylobacter jejuni* Mutants Lacking *_cj_ridA* Have a Motility Defect

**FIGURE 3:**
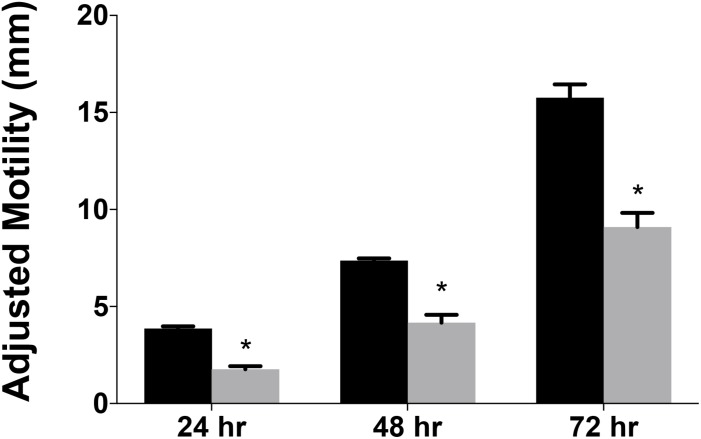
*Campylobacter jejuni ridA* mutants have a significant defect in motility. Swimming motility was determined for *C. jejuni* wild type (black) and a *_cj_ridA* mutant (gray). Ten microliter of cell suspension was inoculated in the center of MH agar (0.4%) plate that was incubated up to 72 h in microaerophilic conditions. Motility was defined as swimming-dependent spread by subtracting the diameter of inoculum diffusion from the motility zone and dividing it by two. Error bars represent the standard errors of the mean of three technical triplicates, for wild type, and two biological replicates in technical triplicate for the *_cj_ridA* mutant. Significance was determined between wild type and*_cj_ridA* mutant for each time period and an asterisk denotes statistically significant (*P* < 0.005) variation between mutants, as determined by an unpaired Student *t* test performed with GraphPad Prism software, v7.0C.

**FIGURE 4:**
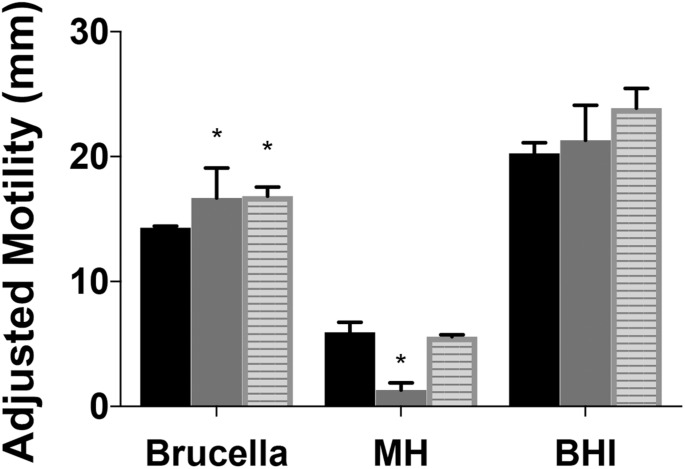
Motility of *Campylobacter jejuni ridA* mutants differs with media composition. Growth (not shown) and motility were improved by complex undefined-media. Motility of the *C. jejuni* wild type (black), *_cj_ridA* mutant (gray), or the chromosomally complemented *_cj_ridA* mutant (*_cj_0046*: *_cj_ridA*-kan) (striped) was improved in Brucella or BHI as compared to MH motility agar (0.4%). Ten microliter of cell suspension was inoculated in the center of each agar (0.4%) plate that was incubated up for 36 h in microaerophilic conditions. Motility was defined as swimming-dependent spread by subtracting the diameter of inoculum diffusion from the motility zone and dividing it by two. Error bars represent the standard errors of the mean of three technical triplicates of wild-type and three biological replicates of *_cj_ridA* and *_cj_0046*:*_cj_ridA*-kan. Significance was determined between wild type and*_cj_ridA* mutant for each time period and an asterisk denotes statistically significant (*P* < 0.02) variation between mutants, as determined by an unpaired Student *t* test performed with GraphPad Prism software, v7.0C.

Data from several bacterial species suggested RidA is involved in flagellar biosynthesis and/or motility (Reuter et al., 2015; Borchert and Downs, 2017a; Irons et al., 2018). A variant of *C. jejuni* 11168 lacking *_cj_ridA* was generated and assessed for swimming motility on Mueller Hinton (MH) medium with 0.4% agar and 0.01% Triphenyltetrazolium Chloride (TTC). The data (Figure 3) showed that the *_cj_ridA* mutant had significantly decreased motility when compared to wild type over the course of 72 h. A non-motile, aflagellate *pseC* mutant was used as a control to determine the spread of the inoculum that was due to diffusion. The motility data in Figure 3 is representative of experiments that were performed more than ten times on ten different days and included three independently constructed *_cj_ridA* mutants. Although there was day-to-day variation in the absolute motility measured, the difference between the mutant and control strains remained consistent at ∼2-fold. Motility was not affected by a lesion in the gene encoding the Rid2 subfamily member (*cj0327*) in either a wild-type background or the *_cj_ridA* mutant, (data not shown). This result supported the conclusion that the _Cj_RidA and Cj0327 proteins are not physiologically redundant in *C. jejuni*, consistent with what has been found in other organisms. Addition of isoleucine (1 mM) to the motility agar did not affect the motility defect of the *_cj_ridA* mutant (data not shown), and motility was restored to wild-type levels when *_cj_ridA* was inserted into pseudogene *cj0046* and expressed with its native promoter (Figure 4).

A previous study reported *_cj_1388* mutants had increased motility compared to wild-type in Brucella motility agar, a rich undefined medium (Reuter et al., 2015). Motility was assessed in Brucella, brain heart infusion (BHI), and MH motility agar for two mutants and wild type and the data are in Figure 4. Both Brucella and BHI media support increased mobility (and growth) of both mutants and wild type. Similar to what was previously reported, *_cj_ridA* mutant motility was 1.1-fold and 1.4-fold higher than wild-type in BHI and Brucella motility media, respectively. Significantly, the *_cj_ridA* mutant displayed a motility defect only on MH medium. The restoration of motility on the two complex media is consistent with regulation of metabolic flux in *_cj_ridA* mutants limiting the production of, and/or damage by, the reactive enamine substrate of the _Cj_RidA protein. For instance, in *S. enterica* and *P. aeruginosa ridA* mutant motility defects only arise when minimal defined media is used and the defects are eliminated by the addition of isoleucine which prevents 2AA formation. Given the complexity of metabolic systems and regulation, minimal defined media will be used in future studies to determine the impact of *_cj_ridA* mutations motility.

### _Cj_RidA Is Required for Full Infection and/or Lysis by Phage NCTC 12673

**FIGURE 5:**
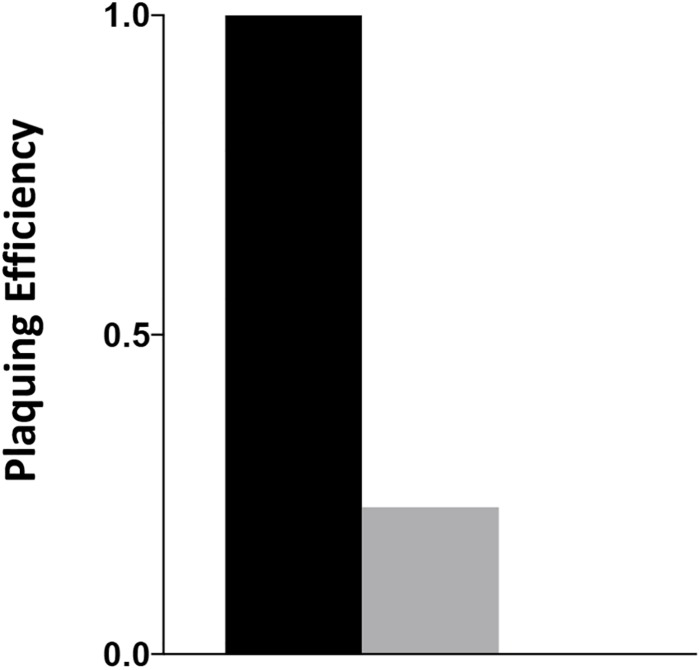
*_cj_ridA* mutants support reduced plaque formation of phage NCTC 12673. A phage lysate was titered on *C. jejuni* wild type (black), a *_cj_ridA* mutant (gray) and *pseC* mutant (plaquing efficiency of zero). Serial dilutions of the lysate were spotted on a NZCYM 0.6% agar overlay seeded with the appropriate bacterial mutant. After 1 day, plaque forming units (PFU/mL lysate) were determined with three technical triplicates of wild type, and two biological replicates in technical duplicate for the *_cj_ridA* mutant. Number of plaques on wild type was defined as an efficiency of 1.

The *C. jejuni* lytic phage, NCTC 12673 has decreased plaquing efficiency on aflagellate mutants (Javed et al., 2015a) and fails to form plaques on *pseC* mutants (Sacher, 2018). Plaque formation by NCTC 12673 was assessed with serially diluted aliquots of a phage lysate spotted on 0.6% agar overlays seeded with the indicated mutant or wild type. The efficiency of plating was tested on wild-type *C. jejuni*, a *_cj_ridA* mutant (Figure 5) and a *pseC* mutant. As expected, no plaques were visible on the *pseC* mutant, which lacks the ability to synthesize pseudaminic acid, the major glycan modification of FlaA and FlaB subunits of the flagellum, and is therefore aflagellate (Javed et al., 2015b; Sacher, 2018). When plated on wild-type *C. jejuni*, the phage titer was 1 × 10^7^ PFU/ml. When the same lysate was plated on a *_cj_ridA* mutant, the titer was 2 × 10^6^. This approximately 5-fold decrease in plating efficiency compared to the parental strain was consistent with the hypothesis that the *_cj_ridA* mutant had a defect in flagellar biosynthesis and/or function.

### *_cj_ridA* Mutants Have a Defect in Autoagglutination

**FIGURE 6:**
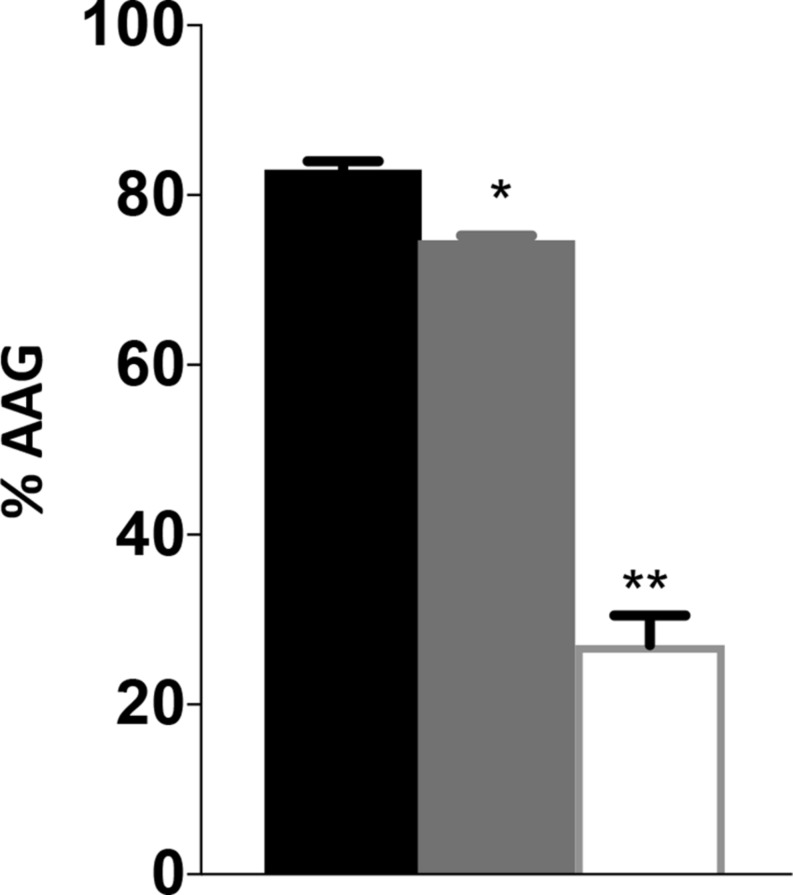
*Campylobacter jejuni ridA* mutants have an autoagglutination defect during the first 24 h. Autoagglutination was measured using *C. jejuni* wild type (black), a *_cj_ridA* mutant (light gray) and a *pseC* mutant (white). % AAG represents the percentage of cells that autoagglutinated and settled in the bottom of the tube after 24 h, determined by the formula [(OD_600__i_–OD_600__r_)/OD_600__i_] × 100. Error bars represent the standard errors of the mean of three technical triplicates, for wild type and *pseC* mutant, and two biological replicates in technical duplicate for *_cj_ridA* mutants. Significance was determined between each mutant and wild type. One asterisk denotes statistically significant (*P* < 0.005) and two asterisks denote statistically significant (*P* < 0.0005) variation between mutants, as determined by an unpaired Student *t* test performed with GraphPad Prism software, v7.0C.

The decreased motility and sensitivity to phage NCTC 12673 suggested a flagellar defect in the *_cj_ridA* mutants. In both cases, the *_cj_ridA* mutant phenotype fell between that of the wild type and the *pseC* mutant, which completely lacks flagella. Consistently, a hallmark of *ridA* mutants is the decreased, but not eliminated activity of the enzymes targeted by 2AA causing phenotypes that are less severe than complete lesions of the relevant enzymes. Changes in autoagglutination (AAG) can also indicate a change in flagella, specifically in flagellar glycan decoration, which correlates with a reduction in virulence (Guerry et al., 2006; Howard et al., 2009; Morrison and Imperiali, 2014). Reuter et al. reported that a *cj1388* (*_cj_ridA*) mutant had a slower rate of AAG compared to wild-type *C. jejuni* 11168, in medium supplemented with Tween-20 (0.002%) (Reuter et al., 2015). AAG was determined in our hands for wild type and *_cj_ridA* mutant after suspension in several different media. Cells were harvested from MH agar plates and suspended in MH or PBS as appropriate. The cell suspension was adjusted to an OD_600_ of 1.0 in 5 mL of: (i) MH, (ii) MH with 0.002% Tween-20, or (iii) PBS. Consistent with previous observations, the *_cj_ridA* mutant had a significant and reproducible decrease in AAG compared to wild type in MH supplemented with 0.002% Tween-20, reflected by more cells remaining in suspension (Figure 6). Each mutant was tested in triplicate. To ensure that any observed difference in phenotype was due to specific mutation, three separate clones for each mutant were tested separately and then the data were combined. The defect of a *_cj_ridA* mutant appeared to reflect a slower rate of AAG, since the defect was significant after a 24-h incubation, but by 48 h the mutants were not significantly different than wild type. In our hands, other media used in reported AAG protocols (PBS or MH alone) failed to result in visible differences between the mutant and wild type. As expected, a *pseC* mutant showed almost complete cessation of AAG, thus another phenotype of the *_cj_ridA* mutant fell between that of a wild type and the *pseC* mutant (Figure 6). The decreased rate of autoagglutination in a *_cj_ridA* mutant supported the emerging model that _Cj_RidA directly or indirectly affects flagellar regulation, biogenesis, glycosylation, or structure.

### Transmission Electron Microscopy Shows _Cj_RidA Impacts Flagella

**FIGURE 7:**
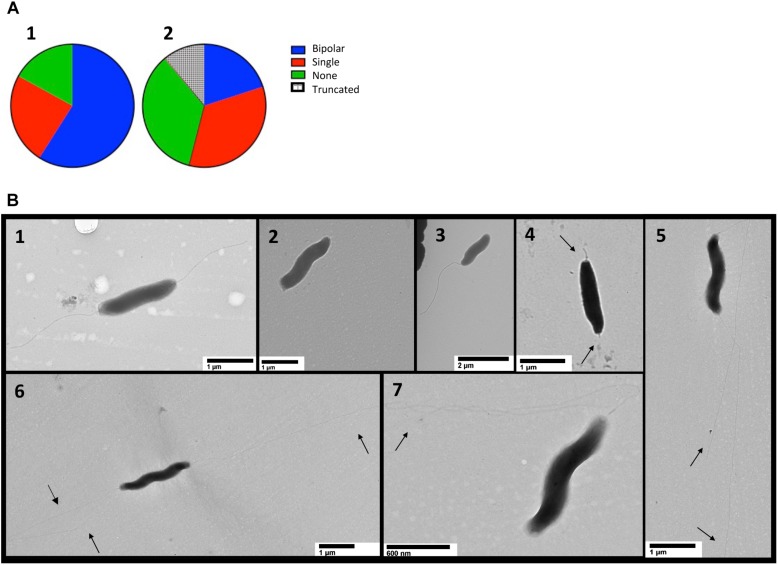
TEM detects flagellar differences in *_cj_ridA* mutants. TEM was used to visualize the flagella of wild type and two *_cj_ridA* mutant*s* on multiple days. One hundred cells with clearly visible poles were assessed in each mutant or wild type. Pie charts in **(A)** represent the distribution of bipolar flagella (blue), a single polar flagellum (red) no flagellum (green) and truncated flagella (hatched). For wild type, *N* = 100, for *_cj_ridA*, *N* = 200 (with 100 from each of two independent mutants) and long and thin flagella (quantified as bipolar or single) were classified by number of flagellar filaments. Lower panels **(B1–B7)** show representative TEM images for cells with: **(B1)** bipolar flagella, wild type is represented: **(B2)** no flagellum, *_cj_ridA* mutant is represented; **(B3)** a single polar flagellum,*_cj_ridA* mutant is represented; **(B4)** truncated flagella, seen only in *_cj_ridA* mutants; and **(B5–B7)** long, potentially thin flagella, seen only in *_cj_ridA* mutant.

Transmission electron microscopy (TEM) was performed on cells harvested from MH agar plates and suspended in PBS (Guerry et al., 2006). Efforts to fix cells with glutaraldehyde and formaldehyde or paraformaldehyde and stain with uranyl acetate or phosphotungstic acid failed to yield clear images and thus the cells were imaged with no fixative or stain (Figure 7B). The number of flagella were quantified using two independently constructed *_cj_ridA* mutants and a wild-type strain of *C. jejuni* (Figure 7A). One hundred cells with two unobstructed poles from each mutant and the wild type were used for quantification. Of the one hundred wild-type cells observed, ∼ 60% had bipolar flagella, ∼20% had a single flagellum, and ∼20% had no visible flagellum. In contrast, of the 200 *_cj_ridA* mutant cells observed, 20% had bipolar flagella, <40% had a single flagellum, <40% had no flagella. Beyond the number, structural anomalies of the flagella were noted in the mutant cells that were not seen in the wild-type sample (Figure 7B). First, there were “nub” structures on one or both poles of the bacterium (∼10% of mutant cells). Secondly, there were instances where flagella in the mutant were unusually long and apparently thinner than the wild type. Together these observations showed that the lack of _Cj_RidA significantly impacted flagellar synthesis and or assembly. TEM images do not provide clarity on the specific flagellar defect caused by a *_cj_ridA* mutation. Regardless, the images, in combination with the phenotypic analysis above allowed the conclusion that _Cj_RidA is important for the full formation of a functional flagella.

### Cj0828 Is the Biosynthetic Serine/Threonine Dehydratase in *C. jejun*i

**FIGURE 8:**
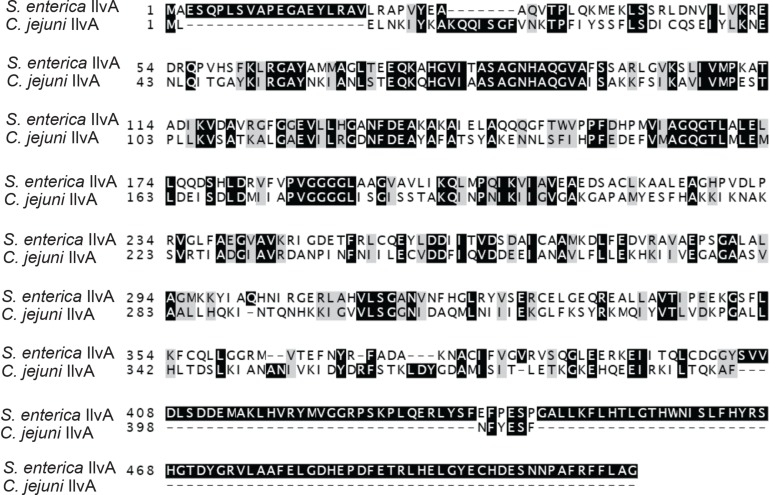
*Campylobacter jejuni* Cj0828 is an IlvA homolog. *Salmonella enterica* IlvA and *Campylobacter jejuni* Cj0828 protein sequences share 32% identity. *Cj0828* (_cj_IlvA) lacks the C-terminal domain that contain the site of allosteric regulation by isoleucine.

In five organisms previously characterized, the phenotypic effects of eliminating the RidA homolog were due to the accumulation of 2AA, generated by a PLP-dependent serine threonine dehydratase enzyme (EC 4.3.1.19). As a consequence, a hallmark of the paradigm thus far has been the suppression of all defects by exogenous isoleucine, which allosterically inhibits the dehydratase enzyme(s). Within this context, it was striking that phenotypes associated with a *_cj_ridA* mutation in *C. jejuni* were apparent in nutrient (MH) medium that contained abundant isoleucine. *C. jejuni* encodes a single gene annotated as a PLP-dependent serine/threonine dehydratase, (Cj0828, EC 4.3.1.19). Cj0828 shares 32% identity to *S. enterica* IlvA (Figure 8) but it lacks the C-terminal domain that contains the allosteric site for inhibition by isoleucine (Gallagher et al., 1998; Chen et al., 2013). These data suggested that if *cj0828* encoded the legitimate biosynthetic threonine dehydratase, the presence of isoleucine would not prevent generation of 2AA by this enzyme. An insertion deletion was introduced into *cj0828* and growth was tested on a defined minimal medium (MCLMAN). In minimal medium, the *cj0828* mutant required isoleucine for full growth, indicating this gene product was the biosynthetic serine/threonine dehydratase *in vivo* (data not shown). To reflect this result, the gene was renamed *_cj_ilvA.* The identification of *_cj_ilvA* suggested three possible scenarios to explain the RidA paradigm in *C. jejuni*: 1) *_cj_ilvA* is constitutively expressed and thus generates 2AA even on nutrient medium, 2) there are other enzyme(s) in the cell that generate 2AA, or 3) 2AA is not responsible for the phenotypes of the *_cj_ridA* mutant. The latter would suggest there was another reactive metabolite produced in the cell that is quenched by _Cj_RidA.

### *C. jejuni* Expands Features of the RidA Paradigm

**FIGURE 9:**
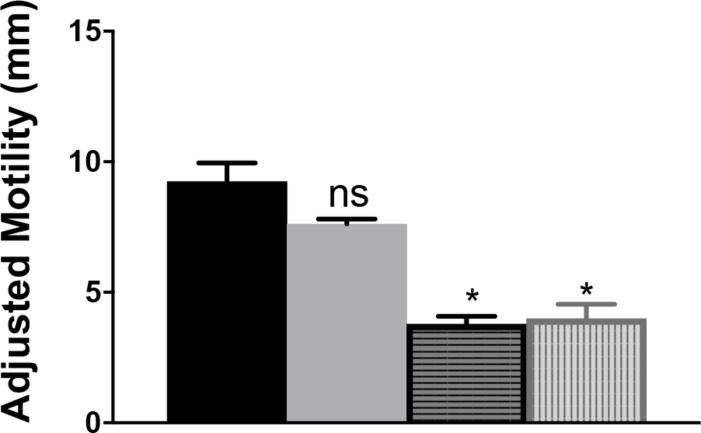
A *_cj_ilvA* mutation does not eliminate the phenotype of a *_cj_ridA* mutant. Mutants were grown on BHI overnight. Then cells were scraped from agar surface, resuspended in 1 mL of PBS and the OD_600_ was set to 1.0. Then, 10 μL of cell suspension was used to inoculate the center of a MH 0.4% agar plate. After 48 h the diameter of motility was measured and the values adjusted to account for the swimming-independent spread of the inoculum, as determined by the non-motile *pseC* mutant. The wild-type parental strain (black) and the *ilvA*:cat mutant (gray) had no defect in motility. The *_cj_ridA* mutant (horizontal stripe) had a >2-fold decrease in motility that was not restored in a*_cj_ridA ilvA* double mutant (vertical stripe). Error bars represent the standard errors of the mean of three technical triplicates, for wild type, and two biological replicates in technical triplicate for *_cj_ridA* and *_cj_ridA cj0828* mutants. Significance was determined between each mutant and wild type; asterisk denotes statistically significant (*P* < 0.0001) variation between mutants, as determined by an unpaired Student *t* test performed with GraphPad Prism software, v7.0C.

The contribution of _Cj_IlvA to the phenotypes of a *_cj_ridA* mutant was tested by constructing a double mutant. A *_cj_ilvA* loss of function mutation was introduced into a _cj_*ridA* mutant background and the resulting double mutant was assessed for motility. Motility assays were performed with the *_cj_ridA _cj_ilvA* double mutant on MH with 0.4% agar (Figure 9). The motility of the *_cj_ilvA* mutant was indistinguishable from the parental wild type. Similarly, the motility of the *_cj_ridA _cj_ilvA* double mutant was no different than the single *_cj_ridA* mutant. Importantly, both mutants carrying a *_cj_ridA* mutation had a >2-fold decrease in motility compared to their respective parental strain. These data showed that _Cj_IlvA was not the source of sufficient 2AA to result in the phenotypes detected for the *_cj_ridA* mutant on MH motility agar. Thus, the source of the reactive enamine presumed to be responsible for the flagellar defects of the *_cj_ridA* mutants remains to be determined.

## Discussion

The data herein demonstrate that the gene designated *cj1388* in *Campylobacter jejuni* 11168 is a RidA with 2AA deaminase activity *in vivo*. *C. jejuni* is the first organism to date where the major phenotypic consequences of lacking RidA are not caused by the activity of a serine threonine dehydratase. Thus *C. jejuni* provides an opportunity to identify additional generators of reactive enamine(s) like 2AA, that can impact the physiology of different organisms in the absence of RidA. One of the two additional 2AA generators found in *S. enterica*, cysteine desulfhydrase (CdsH; EC 2.5.1.47), appears to be present in *C. jejuni* and additional work will determine if this enzyme has a role in generating the phenotypes of a *_cj_ridA* mutant.

Results presented herein, which used three independent *_cj_ridA* mutants, suggest *C. jejuni* 11168 lacking *ridA* has a defect in flagellar biosynthesis, regulation, or structure. *C. jejuni* mutants lacking *_cj_ridA* have defects in motility, AAG, and phage infectivity, all of which require or are enhanced by flagella (Guerry et al., 1991; Golden and Acheson, 2002; Javed et al., 2015b). Motility is essential for *C. jejuni* to move through the viscous mucosal environment to colonize a human host, and protein glycosylation is essential for flagellar biosynthesis and function. Flagellum (FlaA and FlaB) subunits are modified by O-linked pseudaminic and legionaminic acids and their derivatives at up to 19 Ser/Thr sites before export and assembly of the flagellar apparatus (Thibault et al., 2001; Logan et al., 2002; Schirm et al., 2005; Logan, 2006; Ewing et al., 2009). Importantly, thus far the only defined targets of accumulated 2AA are PLP-dependent enzymes. Given the importance of glycosylation of the flagellar subunits, it is possible that the UDP-4-amino-4,6-dideoxy-N-acetyl-ß–L-altrosamine transaminase (Cj1294/PseC; EC 2.6.1.92), a fold-type II PLP-dependent enzyme, could be a critical target of 2AA and thus be damaged in a *_cj_ridA* mutant.

Our favored model suggests that 2AA accumulates in a *_cj_ridA* mutant and damages PLP-dependent enzyme, PseC, leading to a decrease in pseudaminic acid modification on FlaA. Consistent with this model, changes in FlaA glycosylation affect AAG, motility, and virulence (Misawa and Blaser, 2000; Thibault et al., 2001; Logan et al., 2002; Schirm et al., 2005; Guerry et al., 2006; Ewing et al., 2009). Based on other examples, damage by 2AA is expected to reduce the activity of PseC 30–50% (Lambrecht et al., 2010; Ernst and Downs, 2016, 2018; Borchert and Downs, 2017b; Irons et al., 2018). In this case, the phenotypes resulting from PseC damage could vary among the cell population and be similar to the range of phenotypes previously shown from *flaA* point mutations (Ewing et al., 2009; Ulasi et al., 2015; Zebian et al., 2016).

## Materials and Methods

### Bacterial Strains, Plasmids and Media

The strains, plasmids and primers used in this study are listed in Tables 1–3 with their sources. *C. jejuni* human isolate NCTC 11168 (Parkhill et al., 2000) was used as the parental strain. Derivatives of *S. enterica* serovar Typhimurium LT2 (*S. enterica*) were used for *in vivo* complementation studies.

**TABLE 1:** Strains used in this study.

**Organism**	**Mutant ID**	**Genotype**	**Plasmid**	**Source**
*Salmonella enterica*	DM14846	*ridA1*:Tn10(Tc)	pDM1439	Downs lab
	DM14847	*ridA1*:Tn10(Tc)	pCV1 (Empty vector)	VanDrisse and Escalante-Semerena, 2016
	DM16385	*ridA1*:Tn10(Tc)	pDM1577 (*cj1388*)	This study
	DM16513	*ridA1*:Tn10(Tc)	pDM1588 (*cj0327*)	This study
*Escherichia coli*	DM16869	DH5a	pCASO29 (*cj1388*:kan)	Reuter et al., 2015
	DM16508	BL21AI	pDM1589 (pET28b_*cj0327*)	This study
	DM16383	BL21AI	pDM1578 (pET28b_*cj1388*)	This study
*Campylobacter jejuni* 11168	DMC1	Wild type	**–**	Parkhill et al., 2000
	DMC2	*pseC*:kan	**–**	Szymanski Lab
	DMC3	*cjridA*:kan A	**–**	This study
	DMC4	*cjridA*:kan B	**–**	This study
	DMC5	*cjridA*:kan C	**–**	This study
	DMC6	*cj0327*:cat A	**–**	This study
	DMC7	*cj0327*:cat B	**–**	This study
	DMC8	*cj0327*:cat C	**–**	This study
	DMC9	*cjridA*:kan *cj0828*:cat A	**–**	This study
	DMC10	*cjridA*:kan *cj0828*:cat B	**–**	This study
	DMC11	*cjridA*:kan *cj0828*:cat C	**–**	This study
	DMC12	*cj0046*:*cjridA*- cat A	**–**	This study
	DMC13	*cj0046*:*cjridA*- cat B	**–**	This study
	DMC14	*cj0046*:*cjridA*- cat C	**–**	This study

**TABLE 2:** Plasmids used in this study.

**Plasmid**	**Description**	**Source**
pCV1	BspQI modified pBAD24 vector	Downs Lab
pDM1439	*Salmonella enterica ridA* pBAD24	Downs Lab
pDM1577	*Campylobacter jejuni cj1388* pBAD24	This study
pDM1588	*Campylobacter jejuni cj0327* pBAD24	This study
pDM1589	BspQI modified pET28b_*cj0327*	This study
pDM1578	BspQI modified pET28b_*cj1388*	This study
pCASO29	Gene disruption plasmid *cj1388*:kan	Reuter et al., 2015
		

**TABLE 3:** Primers used in this study.

**Purpose**		**Primer name**	**Sequence**
Primers for pBAD24 cloning	Complementation in *Salmonella enterica*	cj1388 pBAD24 For	NNGCTCTTCNTTCATGTCAAACTATCCAAA
		cj1388 pBAD24 Rev	NNGCTCTTCNTTATTATCCTTTTTGAGCGAT
		cj0327 pBAD24 For	NNGCTCTTCNTTCATGATAAAGCGTTTTGA
		cj0327 pBAD24 Rev	NNGCTCTTCNTTATTAATTCTCTCTTAGCTTT
Primers for pET28b protein overexpression		Cj1388_pPET28b_Rev	NNGCTCTTCNGTGTCCTTTTTGAGCGAT
		Cj0327_pET28b_Rev	NNGCTCTTCNGTGATTCTCTCTTAGCTTT
Primers for *Campylobacter jejuni* deletion constructs	Check *cj1388*:kan	cj1388 Seq For	GTTGTTTTTGTCCCTCATCCAT
		cj1388 Seq Rev	TAAAGAAAAAACAATACCTAGC
	Construct *cj0828*:cat	P1 del cj0828	TATTAAACTTCGGAATTTGCTATATTATAACACTTTTTTC
		P2 del cj0828	ATCCACTTTTCAATCTATATCTATTTTTCCTTTGTTTTAAA
		P3 del cj0828	CCCAGTTTGTCGCACTGATAATAACATATACGGCTTAAATA
		P4 del cj0828	CTTCAAAAACAACTTAATAAATTTTCCCATCCTTTTATCC
	Check *cj0828*:cat	cj0828 Seq For	TTTAAAACAAAGGAAAAATA
		cj0828 Seq Rev	TATTTAAGCCGTATATGTTA
	Construct *cj0327*:cat	P1 del Cj0327	AATTTCGGGTTTAAGTTGTA
		P2 del Cj0327	ATCCACTTTTCAATCTATATCTTTATCATGCATATTTTCCT
		P3 del Cj0327	CCCAGTTTGTCGCACTGATAAAATTAAAAATTCTCTCCTCC
		P4 del Cj0327	GGAGACGGTGCAGGTGCTGG
	Check *cj0327*:cat	Cj0327 Seq For	GAATTTCAAGGAAAATATGC
		Cj0327 Seq Rev	ACTTGGGGATCTGCGCTTTT
	Construct *cj0046*:*cj1388*-cat chromosomal complement	P1 Up For Cj0046	GAAGATAATTCTTGGCATTT
		P2 Up Rev Cj0046	TCCACTTTTCAATCTATATCGCAGTATTTGAAGGAGTAAC
		P3 For Cat cassette	GTTACTCCTTCAAATACTGCGATATAGATTGAAAAGTGGA
		P4 Rev Cat cassette	GCCTTTGGATAGTTTGACATGAATTCTCCTTATCAGTGCG
		P5 Cj1388 For	CGCACTGATAAGGAGAATTCATGTCAAACTATCCAAAGGC
		P6 Cj1388 Rev	TAAAACTCCCCTAGCATGTTTTATCCTTTTTGAGCGATGA
		P7 Down For Cj0046	TCATCGCTCAAAAAGGATAAAACATGCTAGGGGAGTTTTA
		P8 Down Rev Cj0046	TTAATAAAATCCTAAAATTTTCC
	Amplify cat cassette	P5 del cj0828	GATATAGATTGAAAAGTGGAT
		P6 del cj0828	TTATCAGTGCGACAAACTGGG

Derivatives of *C. jejuni* 11168 were grown on Mueller Hinton (MH, 21 g/liter), Brain heart infusion (BHI, 37 g/L), Brucella agar (28.1 g/L) or NZCYM (22 g/L) at 37°C under microaerobic conditions (85% N_2_, 10% CO_2_, 5% O_2_) (Blattner et al., 1997). *S. enterica* and *E.* coli strains were grown in Difco Nutrient Broth (8 g/l) with NaCl (5 g/l) at 37°C. Minimal medium was NCE salts with MgSO_4_ (Vogel and Bonner, 1956), trace minerals (Balch and Wolfe, 1976), and 11 mM glucose. Additions, isoleucine (1 mM), and serine (5 mM) were added as indicated. Antibiotic concentrations were as follows; 150 μg/mL ampicillin or 50 μg/mL kanamycin were used for *S. enterica* and 15 μg/mL chloramphenicol or 30 μg/mL kanamycin were used for *C. jejuni*. When needed to induce expression of genes in relevant plasmids, L-arabinose was added (0.2%). Chemicals were purchased from MilliporeSigma (Sigma-Aldrich, St. Louis, MO).

### Growth Quantification

Growth of *S. enterica* in liquid culture was assessed using a BioTek Elx808 microtiter plate reader following optical density at 650 nm at 37°C with slow shaking speed. Overnight cultures of *S. enterica* in biological triplicate were grown in rich medium at 37°C, pelleted and resuspended in an equal volume of sterile NaCl (8.5 g/L). The resulting cell suspension was used to inoculate growth medium (2% inoculum) and growth was monitored for 24 h. The resulting data were plotted using GraphPad Prism 7.0, generating curves in log10-format that display the mean of three replicates and standard deviation of the mean. Specific growth rates (μ) were calculated according to the following equation: ln(X/X_0_)/T, where X is OD_650_, X_0_ is the starting OD_650_ of the exponential growth period monitored, and T is time in hours.

### Molecular Biology

A plasmid (pCASO29) with a deletion/kanamycin insertion construct in *cj1388* was used to construct a *_cj_ridA*:kan mutant (DMC3, DMC4, and DMC5) (Reuter et al., 2015). A *pseC*:kan mutant was obtained from the Szymanski laboratory collection. Additional mutants were constructed using standard methods (Hansen et al., 2007; Tan and Berg, 2004). Briefly, to generate an insertion/deletion in a gene of interest, homology both up- and down-stream to the gene of interest was joined to a drug resistance cassette by overlap extension PCR. PCR products were purified using Qiagen gel extraction kit (ID 28506). The natural competence of *C. jejuni* was exploited to transform the PCR product into cells grown on nutrient rich medium, BHI with 2% yeast extract. After 24 h growth, cells were streaked on selective medium and colonies formed after 3-5 days. Colonies were streaked for isolation and culture stocks were frozen in glycerol. Insertion deletions of relevant genes was confirmed by PCR. The complete protocol for Natural Transformation can be found on Protocols.io at https://dx.doi.org/10.17504/protocols.io.magc2bw.

Derivatives of plasmid pBAD24 and pET28b were created using a BspQI restriction cloning method as previously described by Galloway et al. (2013) with a modified vector that contained the BspQI site (pCV1) (VanDrisse and Escalante-Semerena, 2016). *S. enterica* or *E. coli* competent cells were prepared and transformed using standard method. Transformants were recovered in nutrient broth, plated to selective medium at 37°C before isolating and confirming the plasmid structure.

For *pseC*:km mutant construction, *pseC* was amplified from 11168 using the *pseC*-F and *pseC*-R primers (Table 3). This insert was purified, digested with *Bam*HI and *Xho*I, inserted into PCRscript (Stratagene) digested with the same enzymes, and transformed into *E. coli* DH5α. The extracted plasmids were digested with *Xho*I and *Bam*HI to confirm insert presence and one plasmid subsequently digested with *Nco*I, purified, blunted and treated with alkaline phosphatase to prevent re-ligation. The *pseC* gene was interrupted by ligating a kanamycin resistance cassette (km). The mutant was confirmed using the *pseC*-F/mid KmR primer pair (Table 3) and sequenced.

### Protein Production and Purification

Proteins _Cj_RidA and Cj0327 were purified from *E. coli* strain BL21AI harboring pET vector constructs. The polyhistidine-tagged proteins were purified by nickel-affinity chromatography as previously described (Lambrecht et al., 2010). Overnight cultures in LB (10 mL) were used to inoculate flasks containing super broth (1.5 L) supplemented with kanamycin (50 μg/mL). Cultures were grown at 37°C with shaking until an OD_650_ between 0.7 and 1.0 was reached. Arabinose (0.2%) was added to induce T7 RNA polymerase for 18 h. Cells were harvested by centrifugation at 7,000 × *g* and the cell pellets were stored at −80°C until use. Binding buffer (50 mM potassium phosphate pH 7.5, 100 mM NaCl, 5 mM imidazole, and 10% glycerol) was added to thaw cells (2 mL per gram wet cell weight) along with lysozyme (1 mg/mL) and DNase (20 Units/mL), and the cells were lysed with a One Shot Cell Disruptor at 18,000 psi (Constant Systems). The lysate was clarified by centrifugation (40,000 × *g* for 45 minutes) and passed through a 0.45 μm syringe filter (Argos Technologies) prior to being loaded onto 5 mL HisTrap^TM^ HP column and purified using the manufacturer’s protocol (GE Healthcare). Protein was eluted with a 0–100% gradient of elution buffer (50 mM potassium phosphate pH 7.5, 100 mM NaCl, 500 mM imidazole and 10% glycerol). The fractions were assessed for purity, pooled, and concentrated using a 7,000 molecular weight cut-off protein concentrator (Millipore). The protein preparations were dialyzed into storage buffer (50 mM potassium phosphate pH 7.5, 10% glycerol) using a PD-10 desalting column (GE Healthcare). Proteins were subjected to SDS/PAGE and purity was assessed using a Foto/Analyst FX (Fotodyne) imager and TotalLab Quant v11 densitometry software. Protein concentration was quantified using BCA Protein Assay (Thermo Scientific), and the samples were frozen in liquid nitrogen and stored at −80°C.

### L-Amino Acid Oxidase Assays

The LOX-based assay for Rid activity was adapted from a previously described assay and has been used to assess activity of Rid proteins from several organisms (Hafner and Wellner, 1979; Niehaus et al., 2015; Hodge-Hanson and Downs, 2017; Degani et al., 2018). The 2-iminobutyrate intermediate from 2-aminobutyrate was derivatized with semicarbazide resulting in semicarbazone detected by absorbance at 248 nm. The assay mixture (100 μL total volume) contained potassium pyrophosphate (50 mM, pH 8.7), neutralized semicarbazide (10 mM), bovine liver catalase (1 μg), L-amino acid oxidase (0.5 μg) and 0.1, 1.0 or 10 μM Rid protein. Reactions were started in a 96-well quartz plate with the addition of 2-aminobutyrate to the final concentration of 0.5 mM. Following the addition of substrate, the path length for each well was measured and used along with the molar extinction coefficient for semicarbazone (ε = 10,300 M^–1^ cm^–1^) to calculate the rate of semicarbazone formation. Standard deviation of the mean was determined from three technical triplicates by GraphPad Prism 7.0c.

### Motility

Assays for swimming motility were done by modifying previously described methods (Guerry et al., 1991; Palyada et al., 2009; Neal-McKinney and Konkel, 2012; Vorwerk et al., 2014). Briefly, bacteria were harvested from overnight growth on BHI or MH agar plates into PBS and the OD_600_ was adjusted to 1.0. Ten microliter of the bacteria suspension was inoculated on individual plates by gently piercing the soft agar before expelling the cell suspension into 0.4% agar Brucella, BHI, or MH. Agar plates were incubated at 37°C for 24–72 h. The diameter of each swimming halo was measured and recorded in millimeter (mm). A non-motile *pseC* mutant served as a negative control; the spread of the *pseC* inoculum was subtracted from the motility zone diameter of the experimental strains and the number divided by 2 to get the motility distances as reported in mm. The data shown represent the mean of three technical replicates. For each mutation of interest, three independently isolated mutants were tested to ensure phase variability did not contribute to motility defects. Error bars represent the standard error of the mean. Statistical significance (*P* < *0.02*) was determined by unpaired Student’s test (t test) using GraphPad Prism 7.0c.

### Phage NCTC 12673 Plaque Assay

Plaque formation by NCTC 12673 phage was tested by spotting dilutions of a lysate onto a freshly inoculated bacterial suspension using a standard agar overlay method (Javed et al., 2015a). Briefly, 100 μL of a bacterial suspension (OD_600_ of ∼0.35) was mixed with 5 mL sterile 0.6% molten NZCYM agar (Sigma-Aldrich, St. Louis, MO) and poured onto the surface of a NZCYM plate. After 15 min, 10 μL aliquots of serial dilutions of a phage lysate were spotted onto the agar surface and allowed to completely dry before incubation at 37°C under microaerobic conditions. After 24 h, plaques were counted and the apparent number of PFU/mL was determined.

### Autoagglutination Assays

Published protocols for autoagglutination were adapted for use (Misawa and Blaser, 2000; Guerry et al., 2006; Reuter et al., 2015). Simply, cells were harvested from overnight growth on MH agar plates and resuspended in MH broth. The OD_600_ was measured and adjusted to 1.0 in 5 mL of MH broth with 0.002% Tween-20 in a glass test tube. The top 1 mL was removed and OD_600_ measured (OD_600__i_). The remaining 4 mL sat without shaking at room temperature in air. At 24, and 48 h, a 1 mL aliquot of the liquid was removed and the absorbance was measured to obtain the recorded OD_600_ (OD_600__r_). The percent of autoagglutination (%AAG) reported was calculated as [(OD_600__i_−OD_600__r_)/OD_600__i_] × 100.

## Data Availability

All datasets generated for this study are included in the manuscript and/or the supplementary files.

## Author Contributions

DD and JI conceived the project, designed the experiments, analyzed the data, and wrote the manuscript. JS and CS provided advice on the experimental design and edits in manuscript writing. JI performed the experiments. JI, JS, DD, and CS contributed reagents, materials, and analysis tools.

## Conflict of Interest Statement

The authors declare that the research was conducted in the absence of any commercial or financial relationships that could be construed as a potential conflict of interest.
